# PredSTP: a highly accurate SVM based model to predict sequential cystine stabilized peptides

**DOI:** 10.1186/s12859-015-0633-x

**Published:** 2015-07-05

**Authors:** S. M. Ashiqul Islam, Tanvir Sajed, Christopher Michel Kearney, Erich J Baker

**Affiliations:** 1 Institute of Biomedical Studies, Baylor University, Waco, TX USA; 2 University of Alberta, Edmonton, AB Canada; 3 Department of Biology, Baylor University, Waco, TX USA; 4 Department of Computer Science, Baylor University, One Bear Place #97356, Waco, TX USA

**Keywords:** Machine learning, SVM, Tri-disulfide peptide toxins, Sequential tri-disulfide peptides (STPs), Inhibitory cytine knot (ICKs), Cylotides, Nonknotted STPs, Insecticidal peptides, Antimicrobial peptides

## Abstract

**Background:**

Numerous organisms have evolved a wide range of toxic peptides for self-defense and predation. Their effective interstitial and macro-environmental use requires energetic and structural stability. One successful group of these peptides includes a tri-disulfide domain arrangement that offers toxicity and high stability. Sequential tri-disulfide connectivity variants create highly compact disulfide folds capable of withstanding a variety of environmental stresses. Their combination of toxicity and stability make these peptides remarkably valuable for their potential as bio-insecticides, antimicrobial peptides and peptide drug candidates. However, the wide sequence variation, sources and modalities of group members impose serious limitations on our ability to rapidly identify potential members. As a result, there is a need for automated high-throughput member classification approaches that leverage their demonstrated tertiary and functional homology.

**Results:**

We developed an SVM-based model to predict sequential tri-disulfide peptide (STP) toxins from peptide sequences. One optimized model, called PredSTP, predicted STPs from training set with sensitivity, specificity, precision, accuracy and a Matthews correlation coefficient of 94.86 %, 94.11 %, 84.31 %, 94.30 % and 0.86, respectively, using 200 fold cross validation. The same model outperforms existing prediction approaches in three independent out of sample testsets derived from PDB.

**Conclusion:**

PredSTP can accurately identify a wide range of cystine stabilized peptide toxins directly from sequences in a species-agnostic fashion. The ability to rapidly filter sequences for potential bioactive peptides can greatly compress the time between peptide identification and testing structural and functional properties for possible antimicrobial and insecticidal candidates. A web interface is freely available to predict STP toxins from http://crick.ecs.baylor.edu/.

**Electronic supplementary material:**

The online version of this article (doi:10.1186/s12859-015-0633-x) contains supplementary material, which is available to authorized users.

## Background

Certain proteins are known to be toxic to living organisms [1–3] and this toxicity can serve to provide defense for the host organism against opportunistic insects and microorganisms. In medicine and agriculture, naturally occurring toxic proteins provide an alternative to the rapidly dwindling supply of effective synthetic chemical insecticides, antimicrobials and antifungals [4–7].

Structural stability is critical to the success of these toxic peptides [8]. For example, the physiological environment of an organism contains proteases and highly variable pH which can greatly impact peptide integrity. While a number of approaches can increase the stability of peptides under adverse environments [9, 10], the inclusion of disulfide bonds is one natural way to increase stability [11, 12]. Conversely, in several cases, disulfide bonds may hinder the potent activity of a peptide [13, 14], much work is being undertaken to elucidate disulfide rich stable toxic peptides as insecticides [15, 16], antimicrobial peptides [17] and therapeutic potentials [18, 19].

**Fig. 1:**
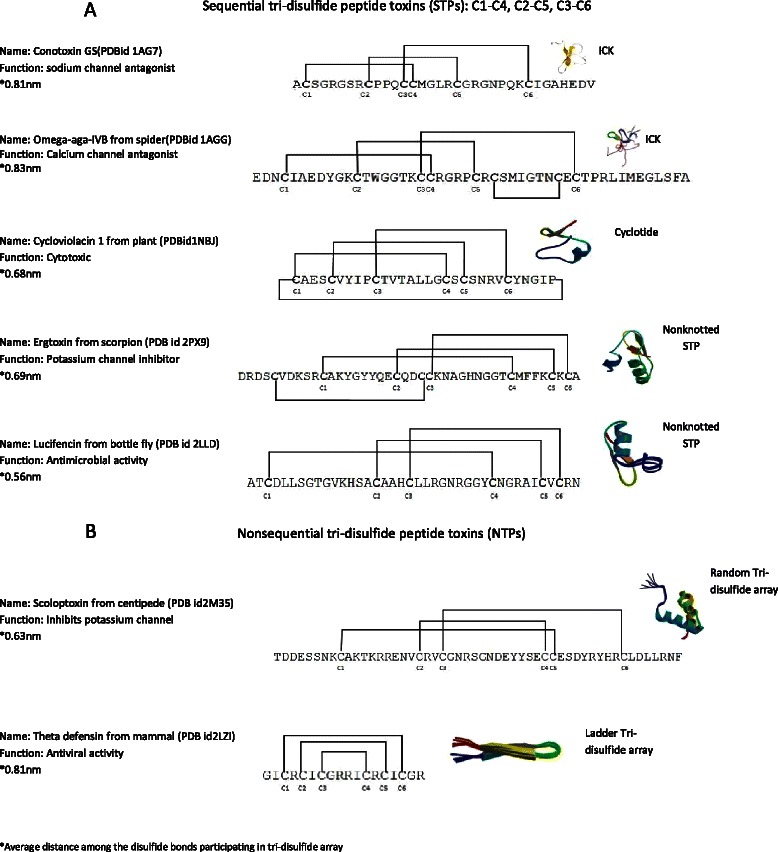
Diagrams of the disulfide connectivity of different cystine stabilized toxic peptides. **a** This figure illustrates the pattern of disulfide connectivity of different types of STP toxins (knotted and non-knotted). Each type is annotated with its name, PDB id, function and jmol estimated average 3D structural distance between disulfide bonds. **b** Illustrates the pattern of disulfide connectivity of NTP toxins with the same type of information

**Fig. 2:**
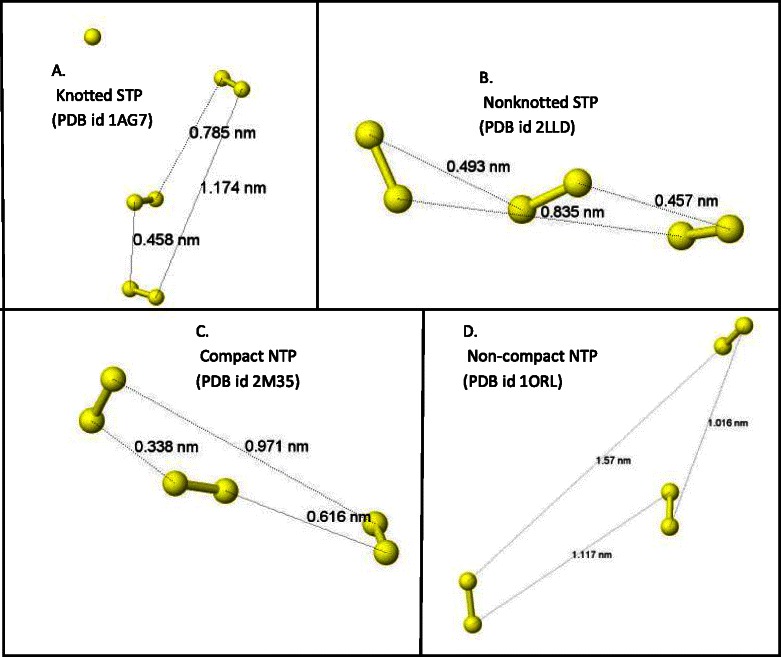
Comparison of the compactness of disulfide bonds in different types of tri-disulfide array containing peptides. Illustration of distances among the non-pairing sulfur molecules participating in the tri-disulfide array. Distances between different sulfur molecule pairs (yellow balls) were measured using jmol software. The mean of these distances indicates the average distance among the disulfide bonds demonstrating the compactness of the tri-disulfide fold in the peptide. **a**, **b**, **c** and **d** show distances of a sample representative of knotted STPs, nonknotted STPs, compact NTPs and non-compact NTPs, respectively, together with their PDB ids. The average of distance in STP toxins (**a** and **b**) is typically less than 0.85 nm, while it is more than 1.2 nm in other tri-disulfide peptides (Non-compact NTPs, data not shown) (**d**). Some NTPs demonstrate a similar compactness (average distance) to STPs and can be designated as compact NTPs (**c**)

Despite a wide range of diversity based on their sources and modes of actions, all cystine stabilized toxins contain a fold with multiple disulfide connectivity [19]. A sequential array of tri-disulfide connectivity is regarded as the most stable [20]. It has a compact cystine trio, where the first cysteine participating in the fold makes a disulfide bond with the fourth cysteine, the second one with the fifth cysteine and the third one with the sixth cysteine (C1–C4, C2–C5, C3–C6). There may be other cysteines in the primary sequence of these peptides, but they do not participate in that sequential tri-disulfide connectivity. This class of proteins includes several large protein families such as knottins [21], scorpion toxin-like superfamily [22], cyclotides [23], and a substantial proportion of diverse peptides comprising antimicrobial peptides and defensins [24]. For clarity, toxic peptides containing this particular stable disulfide connectivity can be referred to as sequential tri-disulfide peptide toxins (STP toxins). Cystine stabilized toxins which do not contain the exact STP bonding array may also offer stability and toxicity [25–28] and can be denoted as nonsequential tri-disulfide peptides (NTPs) (Fig. 1). While STP toxins imply a compact tri-disulfide tertiary confirmation, NTPs toxins may contain both compact or non-compact tri-disulfide folds (Fig. 2).

STP toxins can be further divided into three major groups based on their canonical 3D definitions: Cyclotides [29, 30], inhibitor cystine knots (ICKs) [11] and nonknotted STPs [31–33]. Cyclotides form cyclization through N-C terminus adherence and are renowned as stable peptides containing the sequential tri-disulfide array [34]. In this type of peptide, the third disulfide bond penetrates through the other two disulfide bonds participating in the array and forms a knotted macrocycle of disulfide bonds. ICKs, also known as knottins, are a second type of STPs [35]. They contain the same knotted macrocycle as cyclotides but do not necessarily take the cyclic form. The third type has three sequentially paired disulfide bonds but the third bond does not penetrate the macrocycle, preventing the formation of a ‘knot’. This group may actually contain as many toxins as the first two subgroups combined and includes scorpion toxin-like peptides [22, 33], insect peptides [36], plant peptides [37], and a variety of other peptides. All three STP subgroups are characterized by high stability and toxicity [32, 38–41].

Although STP toxins show similarity in their function and highly constrained folds, they share little sequence identity [11, 31]. As a consequence, discovery of new STPs has traditionally been slow and almost exclusively based on functional properties. In the case of ICKs, an automated discovery process based on sequence similarity using BLAST has previously been paired with sequence and structural algorithms (Knoter 1D and 3D, respectively) to precisely verify knottin candidates [11, 42]. The discovery of knottins via sequence similarity has produced an extensive and well-organized database, despite a scope limited to sequence similarity [25]. Cypred [43] is another relevant software that can predict cyclic proteins and a significant subset of these cyclic peptides have STP like connectivity. While there is no known software to predict non-knotted STPs, there are databases focusing on limited specific families, such as CyBase for cyclotides [44, 45], Conoserver for conotoxins [46] and Arachnoserver for spider toxins [47], but these have little broad application.

Machine learning approaches offer one possible solution for the broad discovery of STP toxins through the use of soft or fuzzy classification schemas, based on salient STP features that extend beyond a reliance on primary sequence similarity. Logic-based machine learning has been used previously to classify the 2D structure of α/α domain type proteins [48], protein-protein interactions [49] or functional classifications of proteins from primary sequence. In particular, Support Vector Machines (SVM), a robust class of machine learning approaches [50], have been successfully used to predict cyclic proteins [43], 2D and 3D protein structures [51, 52] and subcellular localization [53] from primary sequence.

Here, we illustrate a species-agnostic machine learning methodology, called PredSTP (http://crick.ecs.baylor.edu), which is designed to nominate undefined STPs having low sequence identity with currently described STPs. Efficient discovery of new functional members of this class of proteins will enhance our repertoire of potentially stable insecticidal and antimicrobial proteins.

## Methods

### Known STP sequence collection

Sequence of ICKs and cyclotides (knotted STPs) were collected from the Knottin database (http://knottin.cbs.cnrs.fr/) and 167 sequences with solved 3D structures were obtained from this source. An additional 36 sequences of nonknotted STPs with known 3D structures were collected from PDB with 90 % sequence identity (http://www.rcsb.org/, June, 2013). Our total set of 204 candidate sequences (167 from the knottin database and 37 from PDB) were further reduced to remove redundant sequences, defined as sequences sharing ≥ 90 % sequence identity using CD-HIT [54, 55]. A total of 108 sequences were retained from the knottin database set and 36 sequences were from the PDB set, leaving 144 canonical STPs (Additional file 1: Supplement 1). The mean, standard deviation and range of the number of residues in the positive training set are 42.20, 15.70 and 23–143, respectively, with an average number of 6 cysteines per chain.

### Control negative sequence collection

Sequences classified as negative control were collected from PDB using a criterion that was species agnostic and stipulated the exclusion of STPs through positive matches to PDB small proteins (Additional file 1: Supplement 2). 393 sequences were classified as non-STP sequences for the purposes of this study. The mean, standard deviation and range of the number of residues in the chains of the negative training set are 63.16, 25.92 and 9–160, respectively, with an average number of 6 cysteines per chain.

### Independent test sequence collection

Seven independent sets of sequences were collected to verify the robustness of the model (Table 1). Among these were sets classified according to Protein Data Bank (PDB, July 2013) criteria as Eukaryote, Bacteria, Archaea, Virus and Unassigned. In addition, a set of proteins whose sequences were recently solved by NMR and deposited in PDB (July 04, 2012 to March 25, 2014) (NewNMR751) and also the Structural Classification of Protein (SCOP) PDB subset were used (Smallprotein163). Small protein sequences were retrieved with the following parameters: (a) resolution < 1.5 Å, (b) protein chain but not DNA/RNA/Hybrid, and (c) limited to small disulfide rich proteins and have similarity in size, number of disulfide bonds, cystine number and cystine arrangements in their primary structure. The result included STPs, rubredoxins, BPTI-like, snake toxin-like, crambin-like, insulin-like, and high potential iron proteins among others.

**Table 1:** Description of independent test sets analyzed by the new model (PredSTP)

Independent test sample	Query parameters (PDB^a^)	Number of proteins	Number of chains
Small protein 92	○ SCOP: Small Proteins	92	163
	○ Experimental Method: X-RAY		
	○ Resolution: 1.499 or less		
Only Eukaryote	○ TAXONOMY: Eukaryota	45751	102748
Only Bacteria	○ TAXONOMY: Bacteria (eubacteria)	31664	80664
Only Archaea	○ TAXONOMY: Archaea	3127	8366
Only Virus	○ TAXONOMY: Viruses	4629	18642
Unassigned	○ TAXONOMY: Unassigned	479	980
Recently deposited proteins solved by NMR in PDB (July 2012 to March 25 2014)	○ Experimental Method: solution NMR	657	751

^a^PDB date August, 2013 unless otherwise noted. Protein chain types only

### Defining the putative STP cystine motif

STP motifs consist of six cysteine residues (C1–C6) flanked by varying number of non-cysteine residues (Fig. 1). This set of consecutive cysteines is identified here by elucidating the distance between each consecutive pair of cysteines, *i* and *i + 1 as* ∆*C*_*i,i+1*_ (cysteine loops). Based on our global analysis of STP motifs, if the *min*(∆*C*_*i,i+1*_) is greater than three, then the motif is not considered to contain a STP and is discarded (Additional file 1: Figure S1). Likewise, if the *min*(∆*C*_*i,i+1*_) is less than or equal to three and located between C1 and C2 or C2 and C3 the motifs are disregarded as these motifs are often found within electron transport-like proteins such as ferredoxin, rubredoxin, and iron-sulfur proteins [56, 57]. Otherwise, the *min*(∆*C*_*i,i+1*_) was defined to exist between cysteines C3 and C4. This default pair of cysteines is shifted to a higher pair of cysteines if there exist less than 2 additional c-terminus cysteines. For example, if after the default C3 and C4 cysteines are identified, there is only one c-terminus cysteine, then the *min*(∆*C*_*i,i+1*_) is defined as cysteines C4 and C5.

### Proximity Length (P) and Normalized Proximity Length (NP)

After putative STP motifs are identified, a set of three proximity lengths are calculated: *P*_*1*_ = ∆*C*_*1,4*_ *; P*_*2*_ *=* ∆*C*_*2,5*_ *; P*_*3*_ *=* ∆*C*_*3,6*_. Motifs of less than six cysteines, or motifs defined as invalid by our criteria, were assigned *P*_*1*_ = *P*_*2*_ *= P*_*3*_ *=* 0. A Normalized Proximity Length (NP) was then assigned for each proximity length, *P*, resulting in three new values: *NP*_*1*_ *, NP*_*2*_ *,* and *NP*_*3*_. The NP identifies the distance from the observed mean proximity lengths of known STPs to the corresponding bonded cysteines involved in STP cysteine loops in the training set. For example, the average *P* for all STP sequences in the training set is subtracted from the calculated *P* value associated with its corresponding proximity length and normalized as described in *Eq.* 1, where $\overline{x}{P}_j$ is the average of the proximity lengths of known STPs derived from the training set.

1 $$ {\mathrm{NP}}_{\mathrm{j}\in \left\{1,2,3\right\}}=\frac{100}{\left(\left|{P}_j - \overline{\mathrm{x}}{\mathrm{P}}_{\mathrm{j}}\right| + 10\right)} $$

### Detecting least loop length ratio

The least loop length is defined as the *min*(∆*C*_*i,i+1*_) divided by the total length of the peptide. This feature is used as part of feature sets 5 and 6, see Additional file 1: Supplement 3.

### Detecting presence of amino acid between C4–C5 and C5–C6

Data published describing loop lengths of ICKs and cyclotides, which comprise a large subset of STPs [21], motivated a Boolean feature for the presence of inter-loop amino acids. A result of ‘true’ is returned if there is a presence of a minimum of one amino acid in both of the last two loops (C4–C5 and C5–C6) in a putative STP motif.

### Algorithm

We used a Support Vector Machine (SVM) classifier/predictor implementation to elucidate STP toxins. The SVM was implemented using the e1071 library in R (2.15.1). Feature sets were assigned as described in the Additional file 1: Supplement 3*,* and sensitivity, specificity, precision and accuracy were determined after ten-fold cross validation. Initial gamma and cost were set to 0.1 and 0.1, respectively, with the best output at 0.0587. Given 144 STP and 393 non-STP chains, 100 and 300 random samples were chosen, respectively, for a training set over 200 iterations. Feature sets were prioritized based on accuracy.

STP sequences were predicted from the test sets described previously (Table 1) using feature set 6. Due to the limited throughput of the Knoter1D interface, only the “NewNMR751” and “Smallprotein163” (predicted STP chains from the SCOPs derived subset) predictions where compared against Knoter 1D predictions (http://knottin.cbs.cnrs.fr/Tools_1D.php) and validated with Jmol by analyzing the disulfide connectivity using the corresponding PDB files. Results from only the eukaryotic test sets were filtered to remove sequences with ≥ 30 % chain identity and compared against Jmol analysis. Chains exhibiting canonical STP connectivity (C1–C4, C2–C5, C3–C6) were initially considered as true positives. True positives were further cross matched with their PDB annotations to make the final confirmation.

### Confusion matrix creation

A confusion matrix was created to perform the cross validation test. True Positives (TP), False Positives (FP), True Negatives (TN) and False Negatives (FN) were determined from the confusion matrix. Sensitivity [TP/(TP + FN)], specificity [TN/(TN + FP)], precision [TP/(TP + FP)], accuracy [(TP + TN)/(TP + FN + TN + FP)] and Mathews Correlation Coefficient (MCC) [(TPXTN-FPXFN)/sqrt{(TP + FP)(TP + FN)(TN + FP)(TN + FN) were calculated to evaluate the performance of the algorithm.

### PSI BLAST

The BLAST suite (blast-2.2.29+) was installed on a local machine along with the appropriate dataset. The dataset was the chains of proteins deposited in PDB, solved by the NMR method, from July 04, 2012 to March 25, 2014. The selected threshold e-values PSI BLAST [58] were 0.01, 0.1 and 0.5. The number of iterations for PSI BLAST was 5. All other parameters were set as default.

## Results

### Evaluation of feature sets for machine learning outcomes

**Fig. 3:**
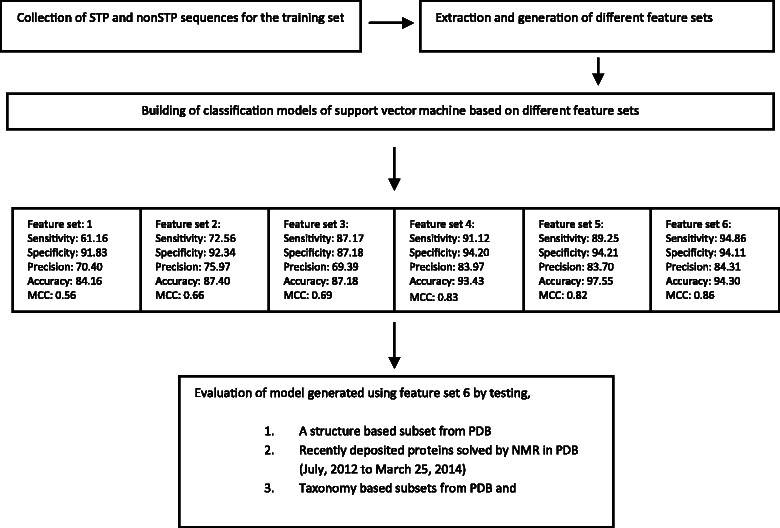
Schematic of the process followed to develop and evaluate the SVM based STP toxin classifier

**Fig. 4:**
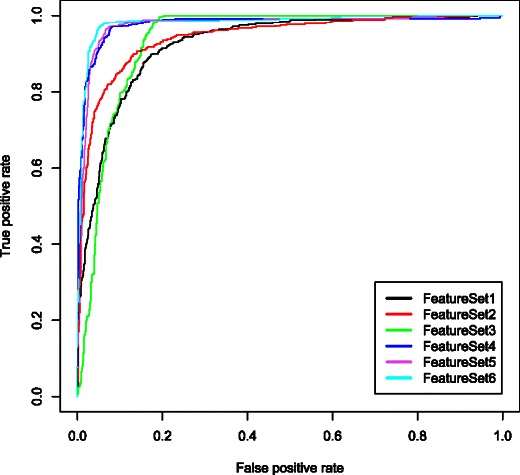
Receiver operating characteristic (ROCR) curves for different models. Receiver operating characteristic curves for the models generated using 6 different feature sets. The area under curve (AUC) generated by feature set 1, 2, 3, 4, 5 and 6 are 0.84, 0.87, 0.87. 0.93, 0.92 and 0.94, respectively

The training data set of 144 STP and 393 non-STP chains was evaluated using randomized sampling over 200 iterations to determine the optimal feature sets. All of the 6 feature sets were examined (Additional file 1: Supplement 3), and the sensitivity, specificity, precision, accuracy and MCC scores were calculated (Fig. 3). Feature set 6 demonstrated the best accuracy and MCC with values of 94.30 %, and 0.86, respectively, and was used for the basis of the remainder of the study. The Receptor Operating Curve (ROC) for feature set 6 is provided in the Fig. 4. In the rest of the article, the model is referred to as PredSTP.

### Classifying STPs from the smallprotein163 subset from PDB

The SmallProtein163 data subset from PDB was analyzed to determine potential automated STP classification. The median residue number of the chains in the Smallprotein163 subset is 54, which is similar to the number of residues in STP chains. In addition, 94 out of the 163 chains contain at least 6 cysteines in their primary sequences. From this subset, PredSTP was able to identify 21 of the 163 potential chains as STP-containing. These putative STP structures were verified by examining their disulfide bonding patterns in Jmol. Of the 21 identified chains by PredSTP, 14 of them were confirmed as true positives (Table 2). An analysis of the 142 negative STP chains predicted by PredSTP demonstrated only one false negative. The sensitivity, specificity, precision and accuracy for this particular dataset were 93.33 %, 99.29 %, 66.66 % and 95.09 %, respectively (Table 3). PDB ids and functions for the positive predicted chains are provided in Additional file 1: Supplement 5.

**Table 2:** Analysis of PredSTP positive hits from smallprotein92 subset

Total PredSTP positive chains	TRUE positive	Knoter1D positive
21	14/21	1/21

**Table 3:** Comparison of evaluation matrices generated by PredSTP using the training set, Smallprotein163 and NewNMR751 subsets from PDB. The confusion matrix generated by PredSTP using the corresponding datasets are provided in Additional file 1: Supplement 4

Source of data	Sensitivity	Specificity	Precision	Accuracy
Training set over 200 iterations	94.86	94.11	84.31	94.30
Smallprotein163	93.33	99.29	66.66	95.09
NewNMR751	91.30	99.72	91.30	99.46

### Testing primary sequences of recently deposited proteins solved by NMR (newNMR 751)

**Fig. 5:**
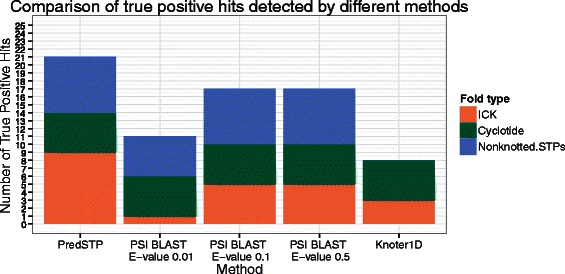
Comparison of the true positive hits detected in newNMR testset using different methods. Bar diagram of a comparison the number of true positive hits detected by testing recently deposited proteins chains solved by NMR in PDB (July, 4 2012 to March, 25 2014) using different methods. Each stack color represents a different type of fold. PredSTP detected nine ICKs, five Cyclotides and six nonknotted STPs; PSI BLAST with E-value 0.01 detected 1 ICK, five Cyclotides and five nonknotted STPs; PSI BLAST with E-value 0.1 and 0.5 detected five ICKs, five Cyclotides and seven nonknotted STPs; Knoter1D detected three ICKs and five Cyclotides

PredSTP was tested against protein sequences with less than 90 % sequence identity and recently solved (July 04, 2012 to March 25, 2014) by NMR. This set of 751 amino acid chains is denoted as newNMR751 and has a median number of 82 residues with 118 chains containing more than six cysteines. The model detected 23 chains from 23 different proteins. Analyzing the disulfide connectivity of the positive hits by Jmol, 21 chains were confirmed as true positive. Based on the number of the predicted outcomes, the sensitivity, specificity, precision and accuracy for this particular dataset were 91.30 %, 99.72 %, 91.30 % and 99.46 %, respectively (Table 3). The true positive chains were further classified into 9 ICKs, 5 cyclotides and 7 nonknotted STPs. PDB ids and functions for positive predictions are provided in Additional file 1: Supplement 6. This set was also analyzed by PSI BLAST [58] and Knoter1D [11]. Knoter1D detected 5 cyclotides, 3 of the 9 ICKs and none of the nonknotted STPs. PSI BLAST (e-value 0.01) detected 12 chains comprising 1 ICK, 5 cyclotides, 5 nonknotted STPs and 1 false positive; PSI BLAST (e-value 0.1) detected 21 chains comprising five ICK, five cyclotides, seven nonknotted STPs and four false positives; PSI BLAST (e-value 0.5) detected 52 chains comprising five ICK, five cyclotides, seven nonknotted STPs and 35 false positives (Fig. 5, Table 4, Additional file 1: Supplement 7).

**Table 4:** Comparison of number of hits detected by different methods in recently deposited proteins solved by NMR in PDB (July 2012 to March 25, 2014)

Method	Positive hits	True positive hits	False positive hits	Calculated sensitivity (%) for STPs^a^	Calculated precision (%) for STPs
PredSTP	23	21	2	91.30	91.30
PSI BLAST with e-value 0.01	13	12	1	52.17	92.30
PSI BLAST with e-value 0.1	21	17	4	73.90	80.95
PSI BLAST with e-value 0.5	52	17	35	73.90	32.69
Knoter1D	8	8	0	57.14	100

^a^Sensitivity for PredSTP and PSI BLAST was calculated based on total experimentally positive STPs (22 chains) in the NewNMR subset from PDB, while sensitivity for Knoter1D was calculated only for Knottins (knotted STPs)

### Evaluation of the PredSTP through scanning and analyzing the Taxonomy subsets from PDB

Finally, after testing the performance of PredSTP against chains from the “SmallProtein163” and “NewNMR751” subsets, which consist of sequences of similar size to the training set, we tested against a set based on diverse taxonomy. We analyzed “Eukaryota”, “Bacteria”, “Viruses”, “Archaea” and “Unassigned” subsets of proteins from the PDB (Table 5). The percentage of positive chains in “Eukaryote” (0.61) is more than the percentage of predicted positive chains for the other three major super kingdoms. In “Eukaryotes”, 636 chains were predicted as STP positive. This number was reduced to 139 chains when chains sharing > 30 % sequence similarity were removed and the first 100 chains (based on PDB id) were manually cross-matched with Jmol analysis to determine true positives. This resulted in a 82 % precision rate (Additional file 1: Supplement 8). In “bacteria”, “virus” and “unassigned” subsets, the precisions were 50 %, 33.33 % and 90 %, respectively (Table 6). In the “Archaea” subset, PredSTP did not predict any potential STP toxins, resulting in no precision. In total, 115 positive hits were analyzed from the “Taxonomy” subset and 93 chains were found as true positive with an overall 80.86 % precision. Individual precision rates for bacteria and viruses were low; this is potentially an artifact of their small sizes. In addition, some bacteria may contain iron-sulfur like transport proteins that mimic STPs by primary structure but are functionally distinct. The number of protein chains containing a minimum of six cysteines and consisting of a maximum 75 residues were also calculated for the same taxonomy subsets from PDB, and the percentages of predicted STPs were 30.08, 6.66, 0, 14.81 and 47.61 for Eukaryotes, bacteria, archaea, virus and unassigned, respectively (Table 7).

**Table 5:** Discovery of STPs across major domains using PDB protein sequence data and PredSTP

PDB subset	Total # of proteins analyzed	Total # of chains	Positive chains predicted by PredSTP	Number of proteins containing positive chains	Percentage of positive chains
Eukaryotes	45751	102748	636	139^a^	0.61
Eubacteria	31664	80664	3	2	0.003
Archaea	3127	8366	0	0	0
Viruses	4629	18642	4	3	0.02
Unassigned	479	980	10	10	1.02

^a^For eukaryotes, 139chains were obtained after screening 636 chains and removing those with ≥ 30 % sequence identity

**Table 6:** Comparison of positive hits detected by PredSTP in different taxonomy based subsets from PDB

PDB subset	PredSTP positive hits	True (structurally) positives	Percent of true positives (Precision)
Eukaryotes	139	82 (100)^a^	82
Bacteria	2	1	50
Archaea	0	0	NA
Viruses	3	1	33
Unassigned	10	9	90
Total	115^a^	93	80.86

^a^For eukaryotes, 100 of the 139 proteins were analyzed in Jmol to find true positives

**Table 7:** Comparison of number of chains (restricted and unrestricted by size) with a minimum of six cysteines to the number of predicted STPs from each domains in PDB

PDB subset	Total # of chains	PredSTP	Type 1 chain	Type 2 chain	Percent of predicted STPs in type 1 chains^a^	Percent of predicted STPs in type 2 chains^b^
Eukayotes	102748	636	2114	32348	30.08	1.96
Bacteria	80664	3	45	9294	6.66	0.03
Archaea	8366	0	6	663	0.00	0
Virus	18642	4	27	3477	14.81	0.11
Unassigned	980	10	21	43	47.61	23.25

^a^Type 1 chain: chains with a maximum of 75 residues and a minimum of six cysteines

^b^Type 2 chain: chains with a minimum of six cysteines regardless chain size

## Discussion

A wide array of toxic peptides, with varying bonding patterns, can be stabilized by disulfide bonds. A large number of these peptides include a sequentially paired disulfide bonding pattern (C1–C4, C2–C5, C3–C6), confirming a compact array of this cystine trio which we refer to here as Sequential Tri-disulfide Peptides (STP). This array includes the well-defined knottin and cyclotide groups that have knotted tertiary structures. They also include a large number of stable toxins that contain the STP bonding pattern but lack the knotted motif typically created by C3–C6 in knottins and cyclotides. Going beyond these groupings, there are other stable toxins that exhibit compact tri-disulfide bonding patterns, but not in the sequentially paired model, including the ladder-type toxins and what we have distinguished as NTPs (Fig. 1).

It is imperative that successful machine learning algorithms select proper training sets and features. We constructed our negative training set with a collection of small proteins verified from the NMR subset deposited in PDB between 2000 and 2010. They contain a similar number of total residues as STPs, and a number have tri-disulfide bonds (NTPs) in their 3D structure. After evaluating several feature sets, a combination of motif-based features and features based on individual amino acids (C, S, H, K, L) generated the best predictions, indicating that differentiation between STPs and nonSTPs lies in both inclusive motifs and primary sequences.

In order to evaluate the performance of PredSTP on out of sample data we developed several independent test sets. The Smallprotein163 and NewNMR751 sets from PDB consist of a substantial number of cysteine rich small proteins. PredSTP showed a better accuracy (95.09 %) for Smallprotein163 than it did for the training set (94.30 %), while the precision was comparatively low (66.66 %). The only STP not detected (PDB id 2C4B) was a heterogonous fusion protein of an STP and a catalytically inactive variant of RNase barnase [59]. On the other hand, a test of performance of PredSTP on the NewNMP751 subset showed an excellent accuracy (99.46 %) with a better precision (90.30 %) than it showed on the training set (Table 3). These results indicate that PredSTP retained its performance when distinguishing STPs from out of sample cysteine rich small proteins.

Knoter1D [21] and Cypred [43] are examples of related software to discover cystine stabilized peptide toxins. Cypred is dedicated for detecting cyclic peptides. Knoter 1D is optimized to identify only knotted STPs using an algorithm that implements BLAST and is dependent on sequence identity with known knotted STPs. This approach does not allow Knoter1D to expand the inclusion of knotted STPs beyond a threshold of sequence identity. However, both knotted and non-knotted STPs vary in their sequences depending on the source organism. To compare our sequence independent algorithm to these approaches, we used the recently deposited protein structure in PDB (NewNMR751). Knoter1D detected only 8 out of 14 knotted STPs (ICKs and cyclotides) and did not detect six new ICKs as they differ significantly from the sequences of the known ICKs (knotted STPs) (Fig. 5). While we compared PredSTP with PSI-BLAST, we used three different E-values to obtain the optimum result from PSI BLAST. Among the three versions, PSI BLAST with E-value 0.1 can detect 21 chains that exhibit the highest sensitivity with a minimum number of 4 false positives. On the other hand, PredSTP detected 21 STPs including the six new ICKs missed by the detection method of Knoter 1D and PSI BLAST. Therefore, in terms of detecting all type of STPs (cyclotides, ICKs and nonknotted STPs), PredSTP demonstrates better sensitivity and precision than PSI BLAST (Table 4).

In order to illustrate the capability of predicting tri-disulfide bonded peptides using PredSTP, we utilized the known paucity of disulfide bonding in bacteria and archaea as compared to eukaryotes [60]. We anticipated a higher proportion of STPs in eukaryotes with respect to the total number of cysteine chains with a maximum of 75 residues and a minimum of six cysteines. The threshold of 75 is chosen because it is well below the length of the longest chain (86 residues long) detected as STP by PredSTP among taxonomy subsets. After testing protein chains from different organismal taxonomy subsets in PDB, we confirmed this by observing that only 6.66 % and 0 % of chains possessing a minimum of six cysteines and maximum 75 residues were predicted as STPs in bacteria and archaea, respectively (Table 7). In contrast, 30 % of the small cysteine-containing chains were predicted as STPs in eukaryotes.

## Conclusion

PredSTP is capable of predicting STP toxins containing a compact tri-disulfide domain and exhibiting identical functional properties in a sequence identity independent manner. Our algorithm implements an automated method to find cystine stabilized toxins containing a compact arrangement of tri-disulfide domain with minimal sequence identity. Therefore, this approach provides useful directions for enhancement of theoretical and experimental research to find new antimicrobial peptides, insecticides and other stable peptide drug candidates by shortening the discovery time of potential bioactive peptides. Further research may benefit from a model that classifies all cystine stabilized peptide toxins (inhibitor or antimicrobial) into the different subgroups based on source, mode of action, and target organisms.

## Additional file

Additional file 1:
**Supplement 1.** PDB ID of control STP chains. **Supplement 2.** PDB ID of control nonSTP chains. **Supplement 3.** Feature Sets Tested for SVM STP prediction. **Supplement 4.** Confusion matrices generated by PredSTP using the training set, Smallprotein163 and NewNMR751 subsets from PDB. **Supplement 5.** List and description of 21 positively predicted proteins in “Smallprotein163” subset from PDB. **Supplement 6.** List and description of 23 positively predicted proteins in NewNMR751 set, deposited in PDB from July 04, 2012 to March 25, 2014. **Supplement 7.** PDB ID of proteins detected by PSI BLAST with different E-values. **Supplement 8.** PDB ids of 100 proteins from the "Eukaryote" subset analyzed manually. **Figure S1.** Distribution of size of the smallest loop lengths of control STP chains from the training set.
