# Supplementary material for: PredSTP: a highly accurate SVM based model to predict sequential cystine stabilized peptides
Source: BMC Bioinformatics. 2015 Jul 5;16:210. doi: 10.1186/s12859-015-0633-x (PMC4491269; doi:10.1186/s12859-015-0633-x)
Supplement: Additional file 1: — Supplement 1. PDB ID of control STP chains. Supplement 2. PDB ID of control nonSTP chains. Supplement 3. Feature Sets Tested for SVM STP prediction. Supplement 4. Confusion matrices generated by PredSTP using the training set, Smallprotein163 and NewNMR751 subsets from PDB. Supplement 5. List and description of 21 positively predicted proteins in “Smallprotein163” subset from PDB. Supplement 6. List and description of 23 positively predicted proteins in NewNMR751 set, deposited in PDB from July 04, 2012 to March 25, 2014. Supplement 7. PDB ID of proteins detected by PSI BLAST with different E-values. Supplement 8. PDB ids of 100 proteins from the "Eukaryote" subset analyzed manually. Figure S1. Distribution of size of the smallest loop lengths of control STP chains from the training set. [file 12859_2015_633_MOESM1_ESM.docx]

**Additional file 1**

**Supplement 1: PDB ID of control STP chains**

>1ACW:A|PDBID

>1AG7:A|PDBID

>1AGG:A|PDBID

>1AGT:A|PDBID

>1AHO:A|PDBID

>1AXH:A|PDBID

>1AYJ:A|PDBID

>1BCG:A|PDBID

>1BH4:A|PDBID

>1BIG:A|PDBID

>1BK8:A|PDBID

>1BKT:A|PDBID

>1BX7:A|PDBID

>1C49:A|PDBID

>1C56:A|PDBID

>1C6W:A|PDBID

>1CHL:A|PDBID

>1CIX:A|PDBID

>1CLV:I|PDBID

>1CMR:A|PDBID

>1CN2:A|PDBID

>1DJT:A|PDBID

>1DKC:A|PDBID

>1DL0:A|PDBID

>1DQ7:A|PDBID

>1EIT:A|PDBID

>1EMX:A|PDBID

>1EYO:A|PDBID

>1F3K:A|PDBID

>1FH3:A|PDBID

>1FJN:A|PDBID

>1FSB:A|PDBID

>1FU3:A|PDBID

>1FYG:A|PDBID

>1G1Z:A|PDBID

>1G9P:A|PDBID

>1GPS:A|PDBID

>1GPT:A|PDBID

>1H20:A|PDBID

>1HA9:A|PDBID

>1HAE:A|PDBID

>1HLY:A|PDBID

>1HP2:A|PDBID

>1HVW:A|PDBID

>1HY9:A|PDBID

>1I26:A|PDBID

>1IE6:A|PDBID

>1IP0:A|PDBID

>1IXT:A|PDBID

>1J5J:A|PDBID

>1JKZ:A|PDBID

>1JLZ:A|PDBID

>1JU8:A|PDBID

>1JXC:A|PDBID

>1K36:A|PDBID

>1K48:A|PDBID

>1KAL:A|PDBID

>1KCP:A|PDBID

>1KOZ:A|PDBID

>1KQI:A|PDBID

>1KV0:A|PDBID

>1L3Y:A|PDBID

>1L4V:A|PDBID

>1LA4:A|PDBID

>1LIR:A|PDBID

>1LMM:A|PDBID

>1LMR:A|PDBID

>1LU0:A|PDBID

>1LUP:A|PDBID

>1M2S:A|PDBID

>1MB6:A|PDBID

>1MCT:I|PDBID

>1MCV:I|PDBID

>1MM0:A|PDBID

>1MMC:A|PDBID

>1MR4:A|PDBID

>1MTX:A|PDBID

>1MVJ:A|PDBID

>1MYN:A|PDBID

>1N8M:A|PDBID

>1NBJ:A|PDBID

>1NH5:A|PDBID

>1NIX:A|PDBID

>1NPI:A|PDBID

>1NRA:A|PDBID

>1OAV:A|PDBID

>1OMY:A|PDBID

>1OZZ:A|PDBID

>1P8B:A|PDBID

>1PE4:A|PDBID

>1PJV:A|PDBID

>1PNH:A|PDBID

>1PT4:A|PDBID

>1PVZ:A|PDBID

>1PX9:A|PDBID

>1Q3J:A|PDBID

>1Q9B:A|PDBID

>1QDP:A|PDBID

>1QK6:A|PDBID

>1QK7:A|PDBID

>1QKY:A|PDBID

>1R1F:A|PDBID

>1R1G:A|PDBID

>1RMK:A|PDBID

>1RYG:A|PDBID

>1SCO:A|PDBID

>1SCY:A|PDBID

>1SEG:A|PDBID

>1SIS:A|PDBID

>1SN4:A|PDBID

>1SNB:A|PDBID

>1SXM:A|PDBID

>1T0Z:A|PDBID

>1TSK:A|PDBID

>1TTK:A|PDBID

>1TTL:A|PDBID

>1TYK:A|PDBID

>1UDK:A|PDBID

>1UGL:A|PDBID

>1UOY:A|PDBID

>1V7F:A|PDBID

>1VB8:A|PDBID

>1VNA:A|PDBID

>1VTX:A|PDBID

>1W7Z:A|PDBID

>1WM7:A|PDBID

>1WM8:A|PDBID

>1WMT:A|PDBID

>1WPD:A|PDBID

>1WQJ:B|PDBID

>1WT7:A|PDBID

>1XDT:R|PDBID

>1Y29:A|PDBID

>1ZNU:A|PDBID

>2A9H:E|PDBID

>2ASC:A|PDBID

>2B3C:A|PDBID

>2BMT:A|PDBID

>2BRZ:A|PDBID

>2C4B:A|PDBID

>2PO8:A|PDBID

>2SN3:A|PDBID

>2UVS:A|PDBID

>2Z3S:A|PDBID

**Supplement 2: PDB ID of control nonSTP chains**

**Defined as > 95% matches to the following PDB Query: “**Experimental method is SOLUTION NMR; SCOP is small proteins; chain type: there is a protein chain but not any DNA or RNA or hybrid; stoichiometry in biological assembly: stoichiometry is MONOMER and TAXONOMY is Eukaryota (eucaryotes) ;released between 2000 and 2010”

>1ADZ:A|PDBID

>1AFP:A|PDBID

>1ANS:A|PDBID

>1APJ:A|PDBID

>1ATA:A|PDBID

>1AW6:A|PDBID

>1B2I:A|PDBID

>1B9G:A|PDBID

>1BBG:A|PDBID

>1BEI:A|PDBID

>1BF0:A|PDBID

>1BGK:A|PDBID

>1BOR:A|PDBID

>1BUS:A|PDBID

>1C2U:A|PDBID

>1C9Q:A|PDBID

>1CCV:A|PDBID

>1CE3:A|PDBID

>1CLD:A|PDBID

>1CO4:A|PDBID

>1COU:A|PDBID

>1CR8:A|PDBID

>1CXW:A|PDBID

>1D2L:A|PDBID

>1D4U:A|PDBID

>1D6G:A|PDBID

>1D6G:B|PDBID

>1DEM:A|PDBID

>1DQC:A|PDBID

>1DTK:A|PDBID

>1DX8:A|PDBID

>1E4U:A|PDBID

>1E88:A|PDBID

>1E8P:A|PDBID

>1E9T:A|PDBID

>1ED0:A|PDBID

>1EFE:A|PDBID

>1F5Y:A|PDBID

>1F81:A|PDBID

>1F8Z:A|PDBID

>1FAQ:A|PDBID

>1FBR:A|PDBID

>1FRE:A|PDBID

>1FVL:A|PDBID

>1FYB:A|PDBID

>1G25:A|PDBID

>1G4F:A|PDBID

>1GKG:A|PDBID

>1GKN:A|PDBID

>1H0Z:A|PDBID

>1H7V:A|PDBID

>1HA8:A|PDBID

>1HCC:A|PDBID

>1HD4:A|PDBID

>1HFH:A|PDBID

>1HFI:A|PDBID

>1HKY:A|PDBID

>1HN6:A|PDBID

>1HPJ:A|PDBID

>1HX2:A|PDBID

>1IGL:A|PDBID

>1IRH:A|PDBID

>1IW4:A|PDBID

>1IYC:A|PDBID

>1IYM:A|PDBID

>1J7M:A|PDBID

>1JC6:A|PDBID

>1JFN:A|PDBID

>1JMN:A|PDBID

>1JMP:A|PDBID

>1JRF:A|PDBID

>1K18:A|PDBID

>1K7B:A|PDBID

>1KBE:A|PDBID

>1KDU:A|PDBID

>1KG1:A|PDBID

>1KGM:A|PDBID

>1KIO:A|PDBID

>1KJ0:A|PDBID

>1KMA:A|PDBID

>1KMX:A|PDBID

>1KS0:A|PDBID

>1KSQ:A|PDBID

>1KUN:A|PDBID

>1L3H:A|PDBID

>1L3X:A|PDBID

>1LD6:A|PDBID

>1LDL:A|PDBID

>1LDR:A|PDBID

>1LPV:A|PDBID

>1M8B:A|PDBID

>1M9O:A|PDBID

>1MGX:A|PDBID

>1MKC:A|PDBID

>1MKN:A|PDBID

>1MPZ:A|PDBID

>1N0Z:A|PDBID

>1N5G:A|PDBID

>1N87:A|PDBID

>1NBL:A|PDBID

>1NJ3:A|PDBID

>1NWV:A|PDBID

>1ORL:A|PDBID

>1OSX:A|PDBID

>1PB5:A|PDBID

>1PCE:A|PDBID

>1PCP:A|PDBID

>1PDC:A|PDBID

>1PK2:A|PDBID

>1PMC:A|PDBID

>1PMX:A|PDBID

>1PMX:B|PDBID

>1PPQ:A|PDBID

>1PS2:A|PDBID

>1PXE:A|PDBID

>1PYC:A|PDBID

>1Q3M:A|PDBID

>1QBH:A|PDBID

>1QGB:A|PDBID

>1QO6:A|PDBID

>1R79:A|PDBID

>1RMJ:A|PDBID

>1RO3:A|PDBID

>1RO4:A|PDBID

>1SHP:A|PDBID

>1SJU:A|PDBID

>1SP7:A|PDBID

>1SRZ:A|PDBID

>1SS3:A|PDBID

>1SSL:A|PDBID

>1SSU:A|PDBID

>1T1H:A|PDBID

>1T50:A|PDBID

>1TBN:A|PDBID

>1TCP:A|PDBID

>1TFI:A|PDBID

>1TFQ:A|PDBID

>1TIH:A|PDBID

>1TOT:A|PDBID

>1TPG:A|PDBID

>1TPM:A|PDBID

>1U34:A|PDBID

>1U5M:A|PDBID

>1UL4:A|PDBID

>1UL5:A|PDBID

>1URK:A|PDBID

>1UUA:A|PDBID

>1UUC:A|PDBID

>1V5N:A|PDBID

>1V87:A|PDBID

>1VD4:A|PDBID

>1VFI:A|PDBID

>1VIB:A|PDBID

>1WD2:A|PDBID

>1WEO:A|PDBID

>1WFE:A|PDBID

>1WFF:A|PDBID

>1WFH:A|PDBID

>1WFL:A|PDBID

>1WFP:A|PDBID

>1WG2:A|PDBID

>1WGE:A|PDBID

>1WGM:A|PDBID

>1WHE:A|PDBID

>1WII:A|PDBID

>1WIM:A|PDBID

>1WJ0:A|PDBID

>1WJ2:A|PDBID

>1WO9:A|PDBID

>1WVK:A|PDBID

>1X4S:A|PDBID

>1XFE:A|PDBID

>1XU6:A|PDBID

>1XUT:A|PDBID

>1YWS:A|PDBID

>1Z60:A|PDBID

>1ZFI:A|PDBID

>2AQA:A|PDBID

>2C6A:A|PDBID

>2CKU:A|PDBID

>2CON:A|PDBID

>2CQE:A|PDBID

>2CR8:A|PDBID

>2CS3:A|PDBID

>2CSV:A|PDBID

>2CT7:A|PDBID

>2D8Q:A|PDBID

>2D8U:A|PDBID

>2D8V:A|PDBID

>2DAN:A|PDBID

>2DID:A|PDBID

>2DIP:A|PDBID

>2DJ8:A|PDBID

>2DJA:A|PDBID

>2DKT:A|PDBID

>2DQ5:A|PDBID

>2ERS:A|PDBID

>2EYA:A|PDBID

>2FC6:A|PDBID

>2FC7:A|PDBID

>2FFT:A|PDBID

>2FN2:A|PDBID

>2GQE:A|PDBID

>2HGF:A|PDBID

>2JQ8:A|PDBID

>2JW6:A|PDBID

>2PJF:A|PDBID

>2UZG:A|PDBID

>3ALC:A|PDBID

>3LRI:A|PDBID

>1ARD:A|PDBID

>1BBO:A|PDBID

>1BHI:A|PDBID

>1FV5:A|PDBID

>1JN7:A|PDBID

>1M36:A|PDBID

>1NCS:A|PDBID

>1NJQ:A|PDBID

>1P7A:A|PDBID

>1PAA:A|PDBID

>1SRK:A|PDBID

>1U85:A|PDBID

>1U86:A|PDBID

>1VA2:A|PDBID

>1VA3:A|PDBID

>1WIR:A|PDBID

>1WJP:A|PDBID

>1WJV:A|PDBID

>1X3C:A|PDBID

>1X5W:A|PDBID

>1X6E:A|PDBID

>1X6F:A|PDBID

>1X6H:A|PDBID

>1XF7:A|PDBID

>1XRZ:A|PDBID

>1ZFD:A|PDBID

>1ZNF:A|PDBID

>1ZR9:A|PDBID

>1ZU1:A|PDBID

>2ADR:A|PDBID

>2COT:A|PDBID

>2CSH:A|PDBID

>2CT1:A|PDBID

>2CT5:A|PDBID

>2CTD:A|PDBID

>2DLK:A|PDBID

>2DLQ:A|PDBID

>2DMD:A|PDBID

>2EPP:A|PDBID

>2EPQ:A|PDBID

>2EPR:A|PDBID

>2EPS:A|PDBID

>2GHF:A|PDBID

>2VRD:A|PDBID

>2VY5:A|PDBID

>2YRK:A|PDBID

>2YT9:A|PDBID

>3ZNF:A|PDBID

>7ZNF:A|PDBID

>1ARD:A|PDBID

>1BBO:A|PDBID

>1BHI:A|PDBID

>1FV5:A|PDBID

>1JN7:A|PDBID

>1M36:A|PDBID

>1NCS:A|PDBID

>1NJQ:A|PDBID

>1P7A:A|PDBID

>1PAA:A|PDBID

>1SRK:A|PDBID

>1U85:A|PDBID

>1U86:A|PDBID

>1VA2:A|PDBID

>1VA3:A|PDBID

>1WIR:A|PDBID

>1WJP:A|PDBID

>1WJV:A|PDBID

>1X3C:A|PDBID

>1X5W:A|PDBID

>1X6E:A|PDBID

>1X6F:A|PDBID

>1X6H:A|PDBID

>1XF7:A|PDBID

>1XRZ:A|PDBID

>1ZFD:A|PDBID

>1ZNF:A|PDBID

>1ZR9:A|PDBID

>1ZU1:A|PDBID

>2ADR:A|PDBID

>2COT:A|PDBID

>2CSH:A|PDBID

>2CT1:A|PDBID

>2CT5:A|PDBID

>2CTD:A|PDBID

>2DLK:A|PDBID

>2DLQ:A|PDBID

>2DMD:A|PDBID

>2EPP:A|PDBID

>2EPQ:A|PDBID

>2EPR:A|PDBID

>2EPS:A|PDBID

>2GHF:A|PDBID

>2VRD:A|PDBID

>2VY5:A|PDBID

>2YRK:A|PDBID

>2YT9:A|PDBID

>3ZNF:A|PDBID

>7ZNF:A|PDBID

>1AQS:A|PDBID

>1DFS:A|PDBID

>1DFT:A|PDBID

>1DMC:A|PDBID

>1DME:A|PDBID

>1FMY:A|PDBID

>1J5L:A|PDBID

>1J5M:A|PDBID

>1JI9:A|PDBID

>1M0G:A|PDBID

>1M0J:A|PDBID

>1MHU:A|PDBID

>1MRT:A|PDBID

>1QJK:A|PDBID

>1QJL:A|PDBID

>1T2Y:A|PDBID

>2MHU:A|PDBID

>2MRB:A|PDBID

>2MRT:A|PDBID

>1F62:A|PDBID

>1FP0:A|PDBID

>1HYI:A|PDBID

>1MM2:A|PDBID

>1MM3:A|PDBID

>1WE9:A|PDBID

>1WEE:A|PDBID

>1WEM:A|PDBID

>1WEN:A|PDBID

>1WEP:A|PDBID

>1WEQ:A|PDBID

>1WES:A|PDBID

>1WEU:A|PDBID

>1WEV:A|PDBID

>1WEW:A|PDBID

>1WFK:A|PDBID

>1WIL:A|PDBID

>2JWO:A|PDBID

>2K1J:A|PDBID

>1CDQ:A|PDBID

>1CHV:S|PDBID

>1COD:A|PDBID

>1CVO:A|PDBID

>1CXO:A|PDBID

>1DRS:A|PDBID

>1ERA:A|PDBID

>1FFJ:A|PDBID

>1G6M:A|PDBID

>1I02:A|PDBID

>1IJC:A|PDBID

>1JE9:A|PDBID

>1JGK:A|PDBID

>1KBS:A|PDBID

>1KS6:A|PDBID

>1LSI:A|PDBID

>1LXG:A|PDBID

>1LXG:B|PDBID

>1MR6:A|PDBID

>1NEA:A|PDBID

>1NTX:A|PDBID

>1PLO:A|PDBID

>1RGJ:A|PDBID

>1RGJ:B|PDBID

>1TFS:A|PDBID

>1TXA:A|PDBID

>1VYC:A|PDBID

>1W6B:A|PDBID

>2CDX:A|PDBID

>1AHL:A|PDBID

>1APF:A|PDBID

>1ATX:A|PDBID

>1B8W:A|PDBID

>1BDS:A|PDBID

>1BNB:A|PDBID

>1E4R:A|PDBID

>1E4T:A|PDBID

>1EWS:A|PDBID

>1FQQ:A|PDBID

>1KJ5:A|PDBID

>1KJ6:A|PDBID

>1SHI:A|PDBID

>1UT3:A|PDBID

>1Z99:A|PDBID

>1ZUF:A|PDBID

>2GW9:A|PDBID

>2JTO:A|PDBID

**Supplement 3: Feature Sets Tested for SVM STP prediction**

We extracted six unique sets of features for use in our machine learning protocol (Supplement Table 6). The first feature set was derived from a multiple sequence alignment (MSA) using MUSCLE[55] in MEGA 5.10. Here, each column was considered an independent feature, providing 318 unique features. Feature sets 2 – 6 were derived from a variety of sequence metadata, including composition and frequency of different amino acids, hydrophobicity, hydrophilicity, neutrality, bonding proximity score (defined below), total length of a chain and least loop to total length ratio (defined below), creating sets of 3, 23, 23, 28 and 28 features, respectively.

**Feature Set 1** Comprises 23 distinct features, derived from calculating the frequency of occurrence of each amino acid plus the *frequency of occurrence* of aggregate hydrophobic (F,Y,L,I,A,M,C,W,V), hydrophilic (R,K,N,D,A,P) and neutral (G,H,S,T,Q) amino acids.

**Feature Set 2**: Comprises 23 distinct features, derived from calculating the number of occurrences of each amino acid plus the *aggregate number of occurrences* of hydrophobic (F,Y,L,I,A,M,C,W,V), hydrophilic (R,K,N,D,A,P), and neutral (G,H,S,T,Q) amino acids.**Feature Set 3:** Comprises three features derived from the Normalized Bonding Distance (NBD) between C1-C4, C2-C5 and C3-C6.

**Feature Set 4**: Comprises 7 distinct features, derived from the Normalized Bonding Distance (NBD) between C1-C4, C2-C5 and C3-C6, Presence of amino acid between C4 -C5 and C5-C6 , presence of double consecutive cysteines in the sequence, total peptide length and the least loop length ratio. The latter was calculated by dividing the length of the shortest ∆Cij by the total length of the peptide.

**Feature Set 5**: Comprises 11 distinct features, derived from Feature set 4 , plus calculating the frequency of occurrences of cysteine, serine, arginine, histindine, lysine (C,S,R,H,K)( plus the *aggregate number of occurrences* of hydrophobic (F,Y,L,I,A,M,C,W,V), hydrophilic (R,K,N,D,A,P), and neutral (G,H,S,T,Q) amino acids.

**Feature Set 6**: Comprises 11 distinct features, derived from Feature set 4 , plus calculating the frequency of occurrences of cysteine, serine, arginine, histindine, lysine (C,S,R,H,K)( plus the *aggregate number of occurrences* of hydrophobic (F,Y,L,I,A,M,C,W,V), hydrophilic (R,K,N,D,A,P), and neutral (G,H,S,T,Q) amino acids.

**Supplement 4: Confusion matrices generated by PredSTP using the training set, Smallprotein163 and NewNMR751 subsets from PDB.**

| Source of data | True positive | True negative | False positive | False negative |
| --- | --- | --- | --- | --- |
| Training set over 200 iterations | 18959 | 56537 | 3463 | 1041 |
| Smallprotein163 | 14 | 141 | 7 | 1 |
| NewNMR751 | 21 | 726 | 2 | 2 |

**Supplement 5: List and description of 21 positively predicted proteins in “Smallprotein163” subset from PDB.**

| PDB ID^1^ | Domain stabilized by tri-disulfide bonds | Disulfide connectivity^2^ | Knoter1D | Function/Class |
| --- | --- | --- | --- | --- |
| *1AHO | Yes | (C1-C4, C2-C5, C3-C6) | No | Scorpion Neurotoxin |
| 1BX7 | Yes | Array is not compact or absent | No | Serine Protease Inhibitor |
| 1BX8 | Yes | Array is not compact or absent | No | Serine Protease Inhibitor |
| *1DJT:A | Yes | (C1-C4, C2-C5, C3-C6) | No | Alpha-like Neurotoxin |
| *1DJT:B | Yes | (C1-C4, C2-C5, C3-C6) | No | Alpha-like Neurotoxin |
| *1KV0:A | Yes | (C1-C4, C2-C5, C3-C6) | No | Alpha-like Toxin |
| *1KV0:A | Yes | (C1-C4, C2-C5, C3-C6) | No | Alpha-like Toxin |
| *1LU0:A | Yes | (C1-C4, C2-C5, C3-C6) | Yes | Hydrolase Inhibitor |
| *1LU0:B | Yes | (C1-C4, C2-C5, C3-C6) | Yes | Hydrolase Inhibitor |
| *1NPI | Yes | (C1-C4, C2-C5, C3-C6) | No | Neurotoxin |
| 1P9G | Yes | C1-C4, C2-C6,C3-C5 | No | Antifungal Protein |
| *1PTX | Yes | (C1-C4, C2-C5, C3-C6) | No | Scorpion Toxin |
| 1R0R | Yes | Array is not compact or absent | No | Serine Protease |
| *1SEG | Yes | (C1-C4, C2-C5, C3-C6) | No | Scorpion Alpha Toxin |
| 1SGP | No | Array is not compact or absent | No | Serine Protease/ Inhibitor |
| *1SN4 | Yes | (C1-C4, C2-C5, C3-C6) | No | Scorpion Neurotoxin |
| *1T7E | Yes | (C1-C4, C2-C5, C3-C6) | No | Alpha-Like Neurotoxin |
| *2ASC | Yes | (C1-C4, C2-C5, C3-C6) | No | Scorpion Toxin |
| 2GKR | Yes | Array is not compact or absent | No | Hydrolase Inhibitor |
| *2SN3 | Yes | (C1-C4, C2-C5, C3-C6) | No | Scorpion Neurotoxin |
| 2UUY | Yes | C1-C3, C2-C6, C4-C5 | No | Tryptase Inhibitor |

^1^. * = true positives

**Supplement 6: List and description of 23 positively predicted proteins in NewNMR751 set,**

**deposited in PDB from July 04, 2012 to March 25, 2014.**

| PDB id | Functional classification | Disulfide connectivity  in the putative motif | True positive | Predicted by Knotter1D |
| --- | --- | --- | --- | --- |
| *2LIX | Potassium channel toxin | C1-C4, C2-C5, C3-C6 | Yes | No |
| *2LJ7 | Antimicrobial Peptide | C1-C4, C2-C5, C3-C6 | Yes | No |
| *2LJS | Cyclotide | C1-C4, C2-C5, C3-C6 | Yes | Yes |
| *2LL1 | Spider toxin | C1-C4, C2-C5, C3-C6 | Yes | Yes |
| *2LN4 | Antimicrobial Peptide | C1-C4, C2-C5, C3-C6 | Yes | No |
| *2LT8 | Antimicrobial Peptide | C1-C4, C2-C5, C3-C6 | Yes | No |
| *2LU9 | Potassium channel toxin | C1-C4, C2-C5, C3-C6 | Yes | No |
| *2LUR | Cyclotide | C1-C4, C2-C5, C3-C6 | Yes | Yes |
| *2LY5 | Defensin-like | C1-C4, C2-C5, C3-C6 | Yes | No |
| *2LZX | New ICK toxin from sponge | C1-C4, C2-C5, C3-C6 | Yes | No |
| *2M2Q | New ICK toxin from bitter melon | C1-C4, C2-C5, C3-C6 | Yes | No |
| *2M2R | New ICK toxin from bitter melon | C1-C4, C2-C5, C3-C6 | Yes | No |
| *2M36 | Spider toxin | C1-C4, C2-C5, C3-C6 | Yes | Yes |
| 2M3H | Apoptotic protein | **Array is not compact or absent | No | No |
| *2M3J | New ICK toxin from sponge | C1-C4, C2-C5, C3-C6 | Yes | No |
| *2M4Z | Spider toxin | C1-C4, C2-C5, C3-C6 | Yes | Yes |
| *2M86 | Cyclotide | C1-C4, C2-C5, C3-C6 | Yes | Yes |
| *2M9O | Cyclotide | C1-C4, C2-C5, C3-C6 | Yes | Yes |
| 2MD7 | Transcription | **Array is not compact or absent | No | No |
| *2MH1 | Cyclotide | C1-C4, C2-C5, C3-C6 | No | Yes |
| *4B2U | New ICK toxin *sicarius spiders* | C1-C4, C2-C5, C3-C6 | Yes | No |
| *4B2V | New ICK toxin *sicarius spiders* | C1-C4, C2-C5, C3-C6 | Yes | No |
| *4BMF | Hydrolase | C1-C4, C2-C5, C3-C6 | Yes | No |

^1^. * = true positives

**Supplement 7: PDB ID of proteins detected by PSI BLAST with different E-values**

PSI BLAST hits with E-value 0.01

>2LJS:A|PDBID

>2LN4:A|PDBID

>2LR3:A|PDBID

>2LT8:A|PDBID

>2LU9:A|PDBID

>2LUR:A|PDBID

>2LY5:A|PDBID

>2M2D:A|PDBID

>2M4Z:A|PDBID

>2M86:A|PDBID

>2M9O:A|PDBID

>2MH1:A|PDBID

PSIBLAST hits with E-value 0.1

>2LIX:A|PDBID

>2LJ7:A|PDBID

>2LJS:A|PDBID

>2LL1:A|PDBID

>2LN4:A|PDBID

>2LR1:A|PDBID

>2LR3:A|PDBID

>2LT8:A|PDBID

>2LU9:A|PDBID

>2LUR:A|PDBID

>2LY1:A|PDBID

>2LY5:A|PDBID

>2LZX:A|PDBID

>2M2D:A|PDBID

>2M2Q:A|PDBID

>2M3J:A|PDBID

>2M4Z:A|PDBID

>2M86:A|PDBID

>2M9O:A|PDBID

>2MBC:A|PDBID

>2MH1:A|PDBID

PSIBLAST hits with E-value 0.5

>2LE8:A|PDBID

>2LGC:A|PDBID

>2LIX:A|PDBID

>2LIY:A|PDBID

>2LJ7:A|PDBID

>2LJS:A|PDBID

>2LL1:A|PDBID

>2LN4:A|PDBID

>2LNC:A|PDBID

>2LR1:A|PDBID

>2LR3:A|PDBID

>2LSQ:A|PDBID

>2LT8:A|PDBID

>2LU9:A|PDBID

>2LUL:A|PDBID

>2LUR:A|PDBID

>2LW3:A|PDBID

>2LWR:A|PDBID

>2LWW:A|PDBID

>2LXF:A|PDBID

>2LY1:A|PDBID

>2LY5:A|PDBID

>2LZO:A|PDBID

>2LZX:A|PDBID

>2M16:A|PDBID

>2M1U:A|PDBID

>2M1X:A|PDBID

>2M2D:A|PDBID

>2M2Q:A|PDBID

>2M3J:A|PDBID

>2M3V:A|PDBID

>2M48:A|PDBID

>2M4E:A|PDBID

>2M4I:A|PDBID

>2M4V:A|PDBID

>2M4Z:A|PDBID

>2M6P:A|PDBID

>2M74:A|PDBID

>2M7P:A|PDBID

>2M86:A|PDBID

>2M9O:A|PDBID

>2MAB:A|PDBID

>2MAB:B|PDBID

>2MBC:A|PDBID

>2ME0:A|PDBID

>2MGX:A|PDBID

>2MH1:A|PDBID

>2MJV:B|PDBID

>2ML5:A|PDBID

>3ZFJ:A|PDBID

>4B2R:A|PDBID

>4BF8:A|PDBID

**Supplement 8: PDB ids of 100 proteins from the "Eukaryote" subset analyzed manually. From 636 chains detected as STP by PredSTP in the Eukaryote dataset, 139 chains were obtained having maximum 30% sequence identity using CD-hit. Out of the 139 chains, the first 100 chains (based on PDB id) were manually analyzed for sequential tri-disulfide bonds using Jmol.**

>1ACW:A|PDBID

>1ADX:A|PDBID

>1AG7:A|PDBID

>1AGG:A|PDBID

>1APQ:A|PDBID

>1ATA:A|PDBID

>1AXH:A|PDBID

>1AYJ:A|PDBID

>1B9G:A|PDBID

>1BBG:A|PDBID

>1BCG:A|PDBID

>1BGK:A|PDBID

>1BMR:A|PDBID

>1BRZ:A|PDBID

>1C4E:A|PDBID

>1C6W:A|PDBID

>1C9P:B|PDBID

>1CCV:A|PDBID

>1CE3:A|PDBID

>1CGI:I|PDBID

>1CHL:A|PDBID

>1CIX:A|PDBID

>1CLV:I|PDBID

>1CMR:A|PDBID

>1CN2:A|PDBID

>1CNN:A|PDBID

>1D1H:A|PDBID

>1DF6:A|PDBID

>1DKC:A|PDBID

>1DS3:I|PDBID

>1ERD:A|PDBID

>1FLE:I|PDBID

>1FU3:A|PDBID

>1G1P:A|PDBID

>1G9P:A|PDBID

>1GL0:I|PDBID

>1GPS:A|PDBID

>1H20:A|PDBID

>1H9H:I|PDBID

>1HD6:A|PDBID

>1HEV:A|PDBID

>1HI7:B|PDBID

>1HLY:A|PDBID

>1HY9:A|PDBID

>1HYK:A|PDBID

>1I26:A|PDBID

>1I2U:A|PDBID

>1IOX:A|PDBID

>1IXT:A|PDBID

>1JJZ:A|PDBID

>1JLZ:A|PDBID

>1JU8:A|PDBID

>1K36:A|PDBID

>1KCP:A|PDBID

>1KLI:L|PDBID

>1KOZ:A|PDBID

>1KQH:A|PDBID

>1L3Y:A|PDBID

>1LMR:A|PDBID

>1LU8:A|PDBID

>1LUP:A|PDBID

>1M2S:A|PDBID

>1MCT:I|PDBID

>1MM0:A|PDBID

>1MM2:A|PDBID

>1MR4:A|PDBID

>1MYN:A|PDBID

>1N89:A|PDBID

>1NE5:A|PDBID

>1NIY:A|PDBID

>1OMC:A|PDBID

>1P9G:A|PDBID

>1PJV:A|PDBID

>1PVZ:A|PDBID

>1Q2K:A|PDBID

>1Q3J:A|PDBID

>1QK7:A|PDBID

>1R1F:A|PDBID

>1RMK:A|PDBID

>1S8K:A|PDBID

>1UDK:A|PDBID

>1UGL:A|PDBID

>1UR6:B|PDBID

>1V91:A|PDBID

>1VIB:A|PDBID

>1WHE:A|PDBID

>1WMT:A|PDBID

>1WQB:A|PDBID

>1X5V:A|PDBID

>1Y29:A|PDBID

>1YP8:A|PDBID

>1YZ2:A|PDBID

>1ZA8:A|PDBID

>1ZAQ:A|PDBID

>1ZFU:A|PDBID

>1ZNT:A|PDBID

>2B68:A|PDBID

>2D56:A|PDBID

>2E2F:A|PDBID

>2E2S:A|PDBID

**Figure S1: Distribution of size of the smallest loop lengths of control STP chains from the training set.**

**
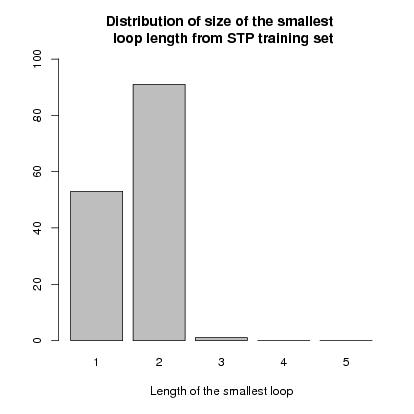
**
